# Developing the Benchmark: Establishing a Gold Standard for the Evaluation of AI Caries Diagnostics

**DOI:** 10.3390/jcm13133846

**Published:** 2024-06-29

**Authors:** Julian Boldt, Matthias Schuster, Gabriel Krastl, Marc Schmitter, Jonas Pfundt, Angelika Stellzig-Eisenhauer, Felix Kunz

**Affiliations:** 1 Department of Prosthetic Dentistry, University Hospital Würzburg, 97070 Würzburg, Germanyschmitter_m@ukw.de (M.S.); pfundt.jonas@stud-mail.uni-wuerzburg.de (J.P.); 2 Center of Dental Traumatology, Department of Conservative Dentistry and Periodontology, University Hospital Würzburg, 97070 Würzburg, Germany; krastl_g@ukw.de; 3 Department of Orthodontics, University Hospital Würzburg, 97070 Würzburg, Germany; stellzig_a@ukw.de (A.S.-E.); kunz_f@ukw.de (F.K.)

**Keywords:** dental caries, artificial intelligence, diagnostics, bitewing, radiography, benchmarking

## Abstract

**Background/Objectives**: The aim of this study was to establish a histology-based gold standard for the evaluation of artificial intelligence (AI)-based caries detection systems on proximal surfaces in bitewing images. **Methods**: Extracted human teeth were used to simulate intraoral situations, including caries-free teeth, teeth with artificially created defects and teeth with natural proximal caries. All 153 simulations were radiographed from seven angles, resulting in 1071 in vitro bitewing images. Histological examination of the carious lesion depth was performed twice by an expert. A total of thirty examiners analyzed all the radiographs for caries. **Results**: We generated in vitro bitewing images to evaluate the performance of AI-based carious lesion detection against a histological gold standard. All examiners achieved a sensitivity of 0.565, a Matthews correlation coefficient (MCC) of 0.578 and an area under the curve (AUC) of 76.1. The histology receiver operating characteristic (ROC) curve significantly outperformed the examiners’ ROC curve (*p* < 0.001). All examiners distinguished induced defects from true caries in 54.6% of cases and correctly classified 99.8% of all teeth. Expert caries classification of the histological images showed a high level of agreement (intraclass correlation coefficient (ICC) = 0.993). Examiner performance varied with caries depth (*p* ≤ 0.008), except between E2 and E1 lesions (*p* = 1), while central beam eccentricity, gender, occupation and experience had no significant influence (all *p* ≥ 0.411). **Conclusions**: This study successfully established an unbiased dataset to evaluate AI-based caries detection on bitewing surfaces and compare it to human judgement, providing a standardized assessment for fair comparison between AI technologies and helping dental professionals to select reliable diagnostic tools.

## 1. Introduction

With the exponential growth in computational power across virtually all semiconductor-based devices, artificial intelligence (AI) is finding its way into medical sciences, driven by the desire to increase diagnostic accuracy, improve treatment outcomes and optimize workflow efficiency [1,2,3]. The increasing prevalence of articles on this subject in literature is evidence of this [4]. From identifying anatomical or pathological structures to assisting with logistical challenges, AI promises to save time and reduce costs [5,6,7].

In human medical imaging, AI applications show promising potential in several areas, particularly in oncology [8]. A major advantage of these AI applications is that their training is based on verified histopathological findings, thus relying on a reliable reference.

In dentistry AI, algorithms have already been developed for automated analysis of radiographs for caries diagnosis [9,10,11,12]. Image recognition in regard to caries detection has been approached using a variety of techniques [13]. However, the traditional comprehensive analysis of X-rays by the dentist is time-consuming and limited by the possibility of human error, which AI promises to largely eliminate [14,15,16,17].

In 2022, Mohammad-Rahimi et al. conducted a systematic review to evaluate the accuracy of automated caries detection systems and showed that the majority of the models included were able to deliver results with clinically acceptable performance parameters, although the quality of studies is often currently low [18]. In particular, in a systematic review and meta-analysis, Ammar and Kühnisch reported acceptable diagnostic accuracy of AI models for caries detection and classification on bitewing radiographs [19]. These radiographs are the most reliable and widely used clinical imaging method for caries diagnosis [20,21]. Despite some promising results, it has also been criticized that AI-based caries diagnostic studies often neither include an appropriate definition of caries nor provide information on the type of carious lesion detected and have limitations in regard to size and heterogeneity of the reported datasets [22,23,24].

The advancement of AI applications for caries detection in bitewing images relies primarily on the use of deep learning networks, primarily convolutional neural networks [25]. This iterative process begins with the compilation of large datasets of annotated bitewing radiographs, in which dental professionals delineate regions of interest corresponding to caries, healthy tooth structure and other anatomical structures [4]. These annotated images are then divided into distinct training and test sets. Using machine learning algorithms, AI-driven methods analyze the training dataset, identifying intricate patterns and extrapolating the desired results [4]. The integrity of the trained model is then evaluated against the separate test dataset, assessing its ability to analyze novel, unseen data. The accuracy of the model is quantified by comparing the predictions derived from the test dataset with the actual annotations. This dichotomy between training and test datasets is crucial to ensure that the AI model goes beyond simply memorizing specific instances from the training dataset, and instead acquires a robust understanding of the general patterns and features that are essential for accurate caries detection.

However, a fundamental limitation arises in the whole training process, which lies in the annotation of radiographs by dentists, representing the AI training gold standard. According to the Standards for Reporting Diagnostic Accuracy Studies (STARD), a gold standard is defined as an error-free reference standard that represents the best available method for determining the presence or absence of the target condition [26]. Although dentists are trained in clinical diagnosis, their sensitivity and specificity for detecting carious lesions on radiographs is somewhat limited [27,28,29,30], in particular for subtle or early stages of lesions. In addition, various factors, such as experience, knowledge, technical skills and time pressure, may influence diagnostic accuracy [31]. While it is undeniable that deep learning can identify features indicative of caries, the underlying methodology has potentially serious practical implications as the predictions only reflect sensitivity and specificity within the training and test data. This concern is exacerbated by the existence of commercial automated dental radiograph analysis software solutions, most of which lack transparency regarding the scientific basis of their AI models.

The aim of this study was, therefore, to develop reliable in vitro simulations of bitewing radiographs based on the histological gold standard to provide a basis for evaluating the performance of AI-based software currently offered by commercial vendors for the automated analysis of caries in bitewing radiographs. In addition, a reference dataset of caries diagnoses from in vitro bitewing radiographs by different examiners was created to serve as a benchmark for predicting whether AI applications can provide a diagnostic advantage to dental examiners.

## 2. Materials and Methods

### 2.1. Ethical Aspects

This study was approved by the Ethics Committee of the Medical Faculty of the University of Würzburg (15/15, 9 February 2015) and was carried out in compliance with the Declaration of Helsinki. All teeth used were extracted for existing clinical indications, with ethical approval, voluntarily and without coercion, and were anonymized. Information provided to patients still allowed for patient withdrawal but excluded the possibility of targeted destruction of donated teeth.

### 2.2. Trial Profile

**Figure 1:**
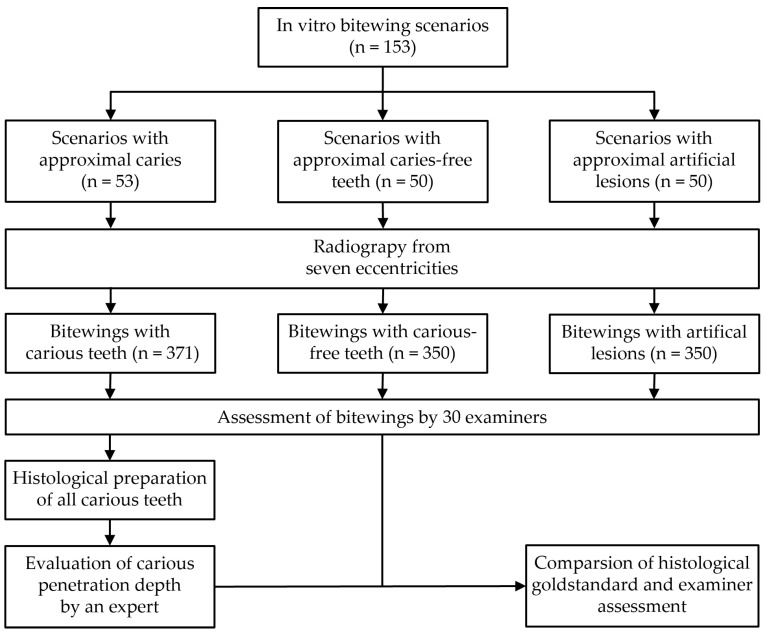
Trial profile.

The trial profile is depicted in Figure 1.

### 2.3. Tooth Selection

**Figure 2:**
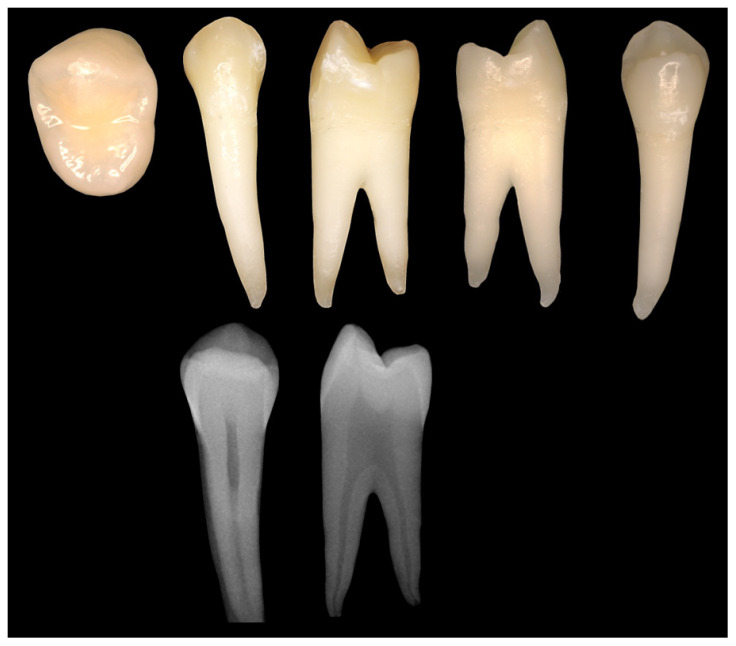
Photographic and radiological documentation of all teeth.

This study used 179 extracted permanent human teeth that were preserved in a 1% tosylchloramide–sodium solution immediately after extraction. All teeth were obtained from various dental clinics and hospitals, ensuring a diverse representation of carious and caries-free conditions. Inclusion criteria were visually and radiographically normal and properly formed permanent teeth with restorative measures that did not significantly interfere with or prevent radiographic caries diagnosis of proximal surfaces. Exclusion criteria comprised completely decayed teeth or root remains, and teeth whose clinical appearance matched hereditary anomalies. All teeth were examined for possible carious lesions by visual inspection using a 2.5× close-up magnification loupe (GTX 2 telescope loupe system; Carl Zeiss Vision GmbH, Aalen, Germany) and tactile examination using a dental probe (EXS3A; Henry Schein Dental Deutschland GmbH, Langen, Germany). A digital single-lens reflex camera (Olympus E-400; Olympus Europa SE & Co. KG, Hamburg, Germany) with 50 mm macro lens (four thirds standard) was used to photograph each tooth from five directions (occlusal, vestibular, oral, mesial, distal). In addition, each tooth was radiographed in the vestibulo-oral and mesiodistal directions (Sirona Heliodent DS; Dentsply Sirona Deutschland GmbH, Bensheim, Germany) (Figure 2). Based on the visual, tactile and radiographic findings, two dentists classified all teeth as carious or caries-free.

### 2.4. Preparation of Artificial Defects

A total of 50 caries-free teeth were used to test the ability to discriminate between carious lesions and artificial defects. The artificial defects were created on the proximal surfaces using 1 mm, 2 mm, 3 mm and 4 mm spherical diamond burs (Gebr. Brasseler GmbH & Co. KG, Lemgo, Germany). During the preparation process, the burs were inserted into the teeth, creating artificial defects half the size of the drill’s diameter.

The selection of diameters ranging from 1 mm to 4 mm was based on findings of Stroud et al. on the mean enamel thickness of permanent posterior teeth [32]. This allowed for clinically accurate lesion simulations.

### 2.5. Bitewing Design

**Figure 3:**
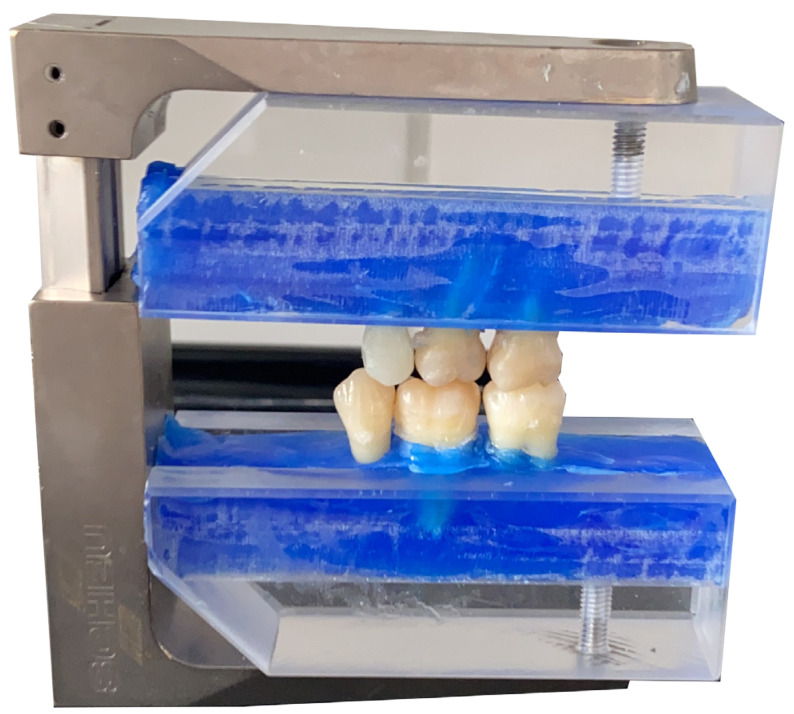
Occlusion holder with fixed teeth simulating clinical bitewing scenarios.

An occlusal holder (Split-Fixator; Scheu-Dental GmbH, Iserlohn, Germany) was fitted with Plexiglas blocks attached at the top and bottom by means of a milled groove. The teeth were embedded in Periphery Wax (Sigma Dental, Handewitt, Germany) and mounted in an anatomically and physiologically accurate configuration to standardize their position for radiographic imaging of the bitewings (Figure 3).

Despite clinical best efforts to use the parallel technique, obtaining superposition-free images of the region of interest in bitewing radiographs remains challenging. Factors such as relative positioning of the teeth, superimpositions, the curvature of the dental arch, the orientation and spatial distortion of the film during exposure and the alignment of the X-ray tube all contribute to the superposition of dental tissue in the interproximal region [33,34]. To mimic clinically relevant situations and improve data quality, the study included not only orthoradial images, but also mesial and distal eccentric images at varying angles. For this purpose, the model was fixed in a rotating vice with a graduated scale that allowed precise angular adjustments in 2-degree increments.

**Figure 4:**
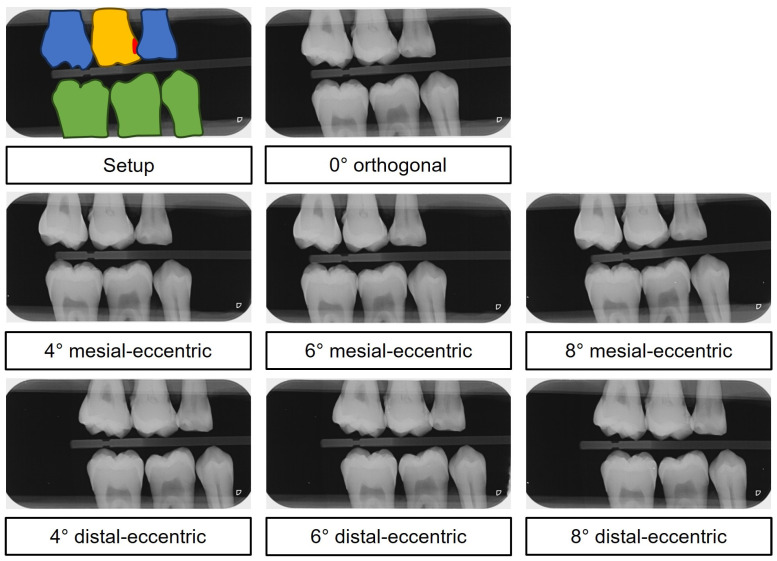
Digital in vitro bitewing images. Top: color-coded setup—yellow: examination tooth, red: carious lesion, blue: adjacent tooth, green: antagonistic tooth. Below: The mesial-eccentric series shows increased superimposition as the ray path becomes increasingly eccentric in the proximal region of teeth 46 and 47. Conversely, the distal-eccentric series shows increased superimposition as the ray path becomes increasingly eccentric in the interproximal region of teeth 15 and 16.

Each examination series yielded a total of seven radiographs, all taken with the same X-ray unit (Sirona Heliodent DS; Dentsply Sirona Deutschland GmbH, Bensheim, Germany; 60 kV, 7 mA, 0,06 ms). These included a 0° orthogonal image and 4°, 6° and 8° mesial and distal eccentric images (totaling 7 images per series) (Figure 4).

### 2.6. Preparation of Histological Samples

**Figure 5:**
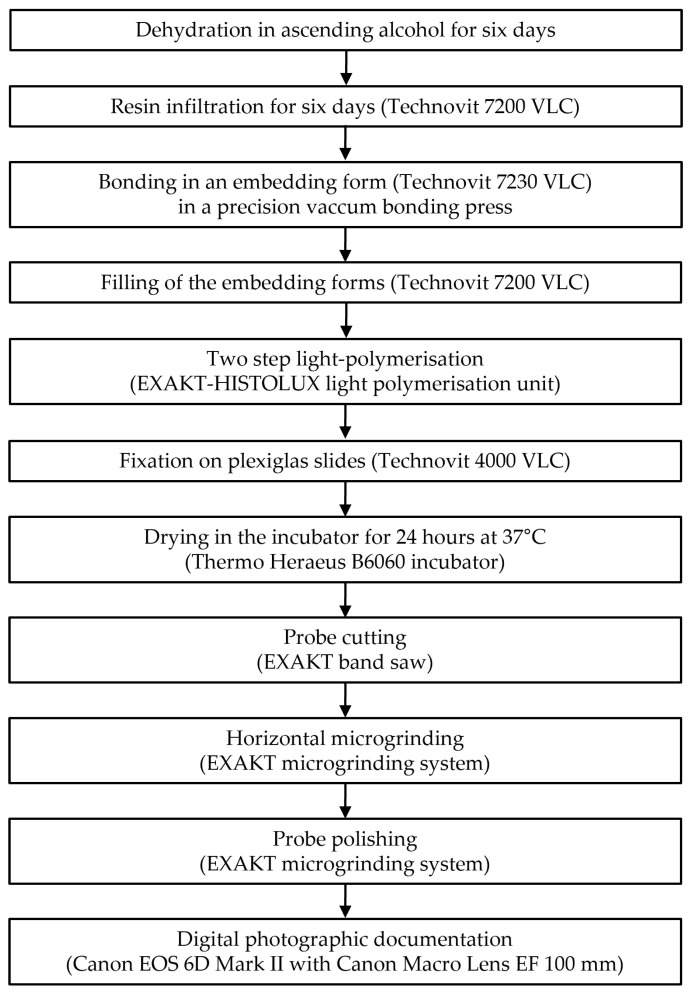
Sample preparation steps.

The sample preparation steps are shown in Figure 5.

After radiography, all carious teeth were subjected to an adapted standardized histological examination procedure (Figure 5) [35]. This was an elaborate process, beginning with a six-day ascending dehydration series with increasing concentrations of ethanol, followed by a six-day resin infiltration (Technovit 7200 VLC; Kulzer GmbH & Co. KG, Wehrheim, Germany) to effectively preserve carious lesions for subsequent processing (Table 1).

The (carious) teeth were then bonded (Technovit 7230 VLC; Kulzer GmbH & Co. KG, Wehrheim, Germany), vestibular side down, to an embedding form (Kulzer GmbH & Co. KG, Wehrheim, Germany) using a disposable spatula and cured with UV light for 10 min in a precision vacuum bonding press (EXAKT Apparatebau GmbH & Co. KG, Norderstedt, Germany). The forms were filled with embedding resin (Technovit 7200 VLC; Kulzer GmbH & Co. KG, Wehrheim, Germany) using a disposable pipette. Pre-polymerization was performed in an EXAKT-HISTOLUX light polymerization unit (Exakt Apparatebau GmbH & Co. KG, Nordstedt) with two UV lamps for two hours, followed by the actual polymerization with eight UV lamps for a further eight hours. The polymerized blocks were fixed to Plexiglas slides (Walter-Messner GmbH, Oststeinbek, Germany) using mixed Technovit 4000 (Kulzer GmbH & Co. KG, Wehrheim, Germany) and cured with UV light for 10 min in the precision vacuum bonding press (EXAKT Apparatebau GmbH & Co. KG, Norderstedt, Germany). Before further processing, the samples were dried in an incubator (Thermo Heraeus B6060 incubator; Heraeus Holding GmbH, Hanau, Germany) for 24 h at 37 °C.

The (carious) teeth were sectioned directly in front of the lesion using a saw with a diamond-coated band 100 µm wide (EXAKT Apparatebau GmbH & Co. KG, Norderstedt, Germany) under constant water cooling. Due to inherent vibrations and the cutting width of the saw blade, a loss of tooth substance of approximately 300 µm per cut (slice) was assumed. During the cutting process, the block was fixed to the machine by a vacuum pump at 680 mbar and pulled through the saw blade by a constant force of 400 g (40 N). The hard-cut method was used to divide the carious teeth before the lesion reached its maximum extent.

**Figure 6:**
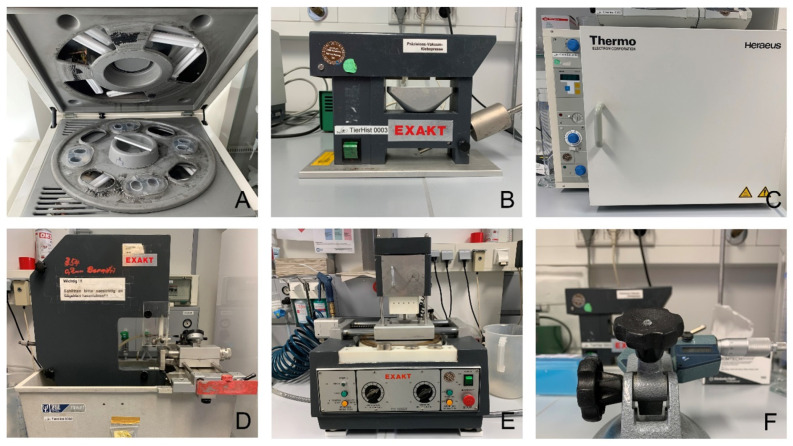
Equipment for the preparation of histological specimens. (**A**): EXAKT-HISTOLUX light polymerization unit, (**B**) EXAKT precision vacuum bonding press, (**C**): Thermo Heraeus B6060 Incubator, (**D**): EXAKT band saw, (**E**): EXAKT horizontal microgrinding system, (**F**): EXAKT micrometer screw.

This was followed by a meticulous, progressive approach to the carious defect using the wet grinding technique with the EXAKT horizontal microgrinding system and a 400 g press weight (EXAKT Apparatebau GmbH & Co. KG, Norderstedt, Germany) (Figure 6). The microgrinding unit was calibrated by grinding a microscope slide with 1200 grit Al_2_O_3_ sandpaper for two minutes. A difference in the slide of no more than 5 μm at four different measuring points was considered acceptable. The final step was polishing with the EXAKT horizontal microgrinding system using 2400 and 4000 grit Al_2_O_3_ sandpaper, with each incremental step documented by digital photographic records with a digital single-lens reflex camera (Canon EOS 6D Mark II; Canon Deutschland GmbH, Krefeld, Germany) and a macro lens (Canon Macro Lens EF 100 mm, Canon Deutschland GmbH, Krefeld, Germany) to illustrate the maximum extent of the lesion in the mesiodistal direction. The removal of tooth material between grinding was determined by measuring the thickness with a micrometer screw (EXAKT Apparatebau GmbH & Co. KG, Norderstedt, Germany).

### 2.7. Lesion Classification of the Histological Samples

**Figure 7:**
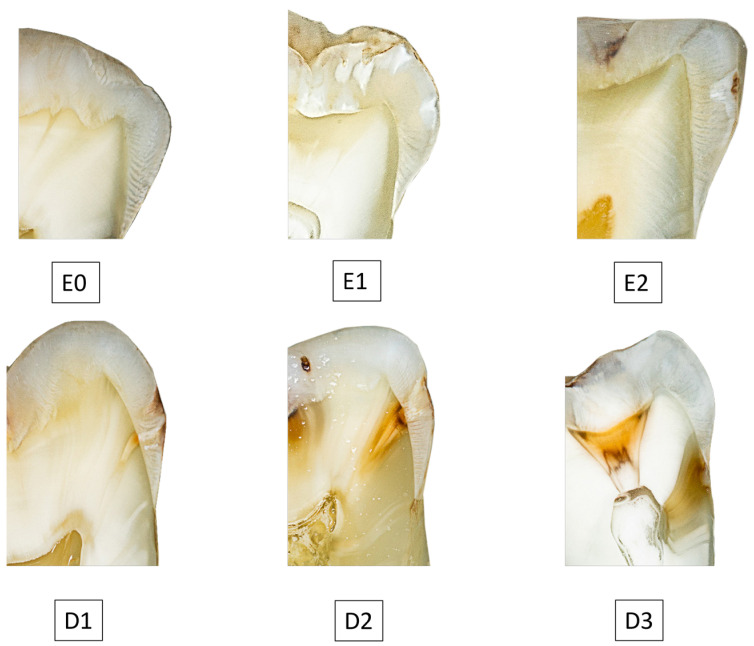
Histological specimen with different proximal carious lesion depths. E0 = Caries-free, E1 = Caries limited to the outer half of the enamel, E2 = Caries extending to the inner half of the enamel, D1 = Caries in the outer third of dentin, D2 = Caries in the middle third of dentin, D3 = Caries in the dentinal third close to the pulp or up to the pulp.

All histological specimens, with the maximum extent of the carious lesion in mesio-distal direction, were digitally photographed and displayed on a diagnostic monitor (Nio Color 2 MP LED; Barco, Kortrijk, Brussels, Belgium) with no time limit (Figure 7). A review was performed twice at three-month intervals by an expert with extensive professional and scientific experience, following the common radiographic classification scheme (Table 2).

The characteristics of the histological analysis are summarized in Table 3.

### 2.8. Radiographic Caries Diagnostic by Dental Examiners

To benchmark dental examiners when analyzing in vitro bitewing images, 10 clinicians, 10 private practitioners and 10 students were asked to evaluate these radiographs.

Clinicians were defined as dentists providing care in a hospital setting, whereas private practitioners were defined as dentists working independently outside an institutional setting, usually in their own private practice. As a baseline, all participants were informed that all teeth would be examined for the presence or absence of proximal caries. Each participant evaluated a random selection of 35 to 36 bitewing images on a dental diagnostic monitor (Nio Color 2 MP LED; Barco, Kortrijk, Brussels, Belgium) without a time limit. All examiners were categorized according to gender, occupation and professional experience to assess respective influence on the quality of caries findings in bitewing radiographs.

### 2.9. Statistical Analysis and Performance Metrics

Statistical analyses were performed using R (version 4.3.2). Quality of carious lesion classification was determined by assessing the intrarater reliability using the intraclass correlation coefficient (ICC). The performance of the combined examiners was assessed using several metrics, including sensitivity, specificity, accuracy, positive and negative predictive values (PPV/NPV), area “under the curve” (AUC), F1 score and Matthews correlation coefficient (MCC).

The F1 score, a harmonic mean of precision and sensitivity, is a commonly used metric for binary classifier evaluation and ranges from 0 to 1, with higher values indicating superior classifier performance. It is defined as $2\times \frac{\left(\mathrm{PPV}\times \mathrm{sensitivity}\right)}{\left(\mathrm{PPV}+\mathrm{sensitivity}\right)}$.

The Matthews Correlation Coefficient (MCC) is another key parameter for evaluating predictions against actual values and provides a reliable assessment of performance. The MCC is defined as $\frac{\left(\mathrm{TN}\times \mathrm{RP}\right)-(\mathrm{FN}\times \mathrm{FP})}{\sqrt{\left(\mathrm{TP}+\mathrm{FP}\right)\times (TP+\mathrm{FN})\times \left(\mathrm{TN}+\mathrm{FP}\right)\times (\mathrm{TN}+\mathrm{FN})}}$. An MCC value of 1 indicates a perfect prediction, while −1 indicates a complete disagreement between prediction and observation, and 0 indicates a random prediction. By including true negatives, false positives, false negatives and true positives, the MCC provides a comprehensive assessment of the predictive accuracy of the system or examiner under investigation.

De Long’s test was used to compare the receiver operating characteristic (ROC) curves of histology and examiners. In addition, MCC scores were tested for differences in correlation using Bonferroni correction to compare performance across varying eccentricities of the central X-ray beam, the different carious lesion depths, gender, occupation and experience. The ability of the examiners to discriminate between artificially induced defects and true caries was investigated by comparing correct and incorrect predictions.

### 2.10. Sample Sice Planning

Our sample size planning was based on the number of bitewing radiographs required for accurate and reliable AI-assisted caries detection. We reviewed recent studies in this area and found that the number of bitewing radiographs used ranged from 45 to 252, with an average of 114 [9,36,37,38,39,40]. Due to the wide variation in the number of bitewing radiographs used in the literature, we used significantly more radiographs for testing in our study, with a total of 371 bitewing radiographs of 53 carious teeth. It can, therefore, be concluded that our sample size provides a robust dataset for evaluation.

## 3. Results

### 3.1. Examiner Characteristics

The metrics for all examiners are shown in Table 4. Private practitioners, clinicians and students were equally represented with ten examiners each. The private practitioners were almost equally divided between six examiners with less than five years’ experience and four examiners with five or more years’ experience. However, there was some imbalance between the two groups, with four male and six female private practitioners. All ten clinicians were evenly split between those with less than five years’ experience and those with five or more years’ experience, as was the gender split with five males and five females. There were eight female students compared to two males. Eight of the eleven examiners with less than five years’ experience were male, followed by three female examiners in this group. In the group of nine examiners with five or more years’ experience, there were three male and six female examiners. Of the thirty examiners, thirteen were male and seven were female.

### 3.2. Reliability of Histological Lesion Classification

Intrarater reliability was very high throughout both assessment rounds (ICC: 0.993; 95%-CI [0.990; 0.995]). In two cases where the expert’s categorization of carious lesions differed between the two rounds of examination, a second expert was consulted to determine the final lesion class.

### 3.3. Examiners Performance Metrics

All examiners reached a combined accuracy of 0.799, a sensitivity of 0.565, a specificity of 0.956, a PPV of 0.896, a NPV of 0.765, an AUC of 76.1, a F1 score of 0.693 and a MCC of 0.578 (Table 5).

### 3.4. AUC

**Figure 8:**
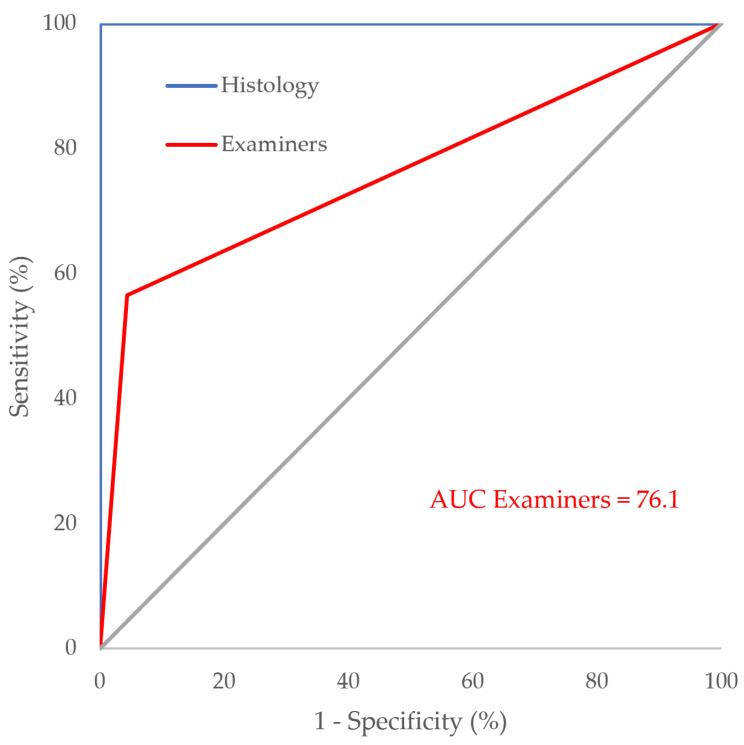
AUC of examiners and histology.

All examiners achieved a combined AUC of 76.1, whereas histology, serving as the gold standard method in caries diagnostic research, was assigned an AUC of 100 (Figure 8). Statistical analysis using De Long’s test to compare the two ROC curves revealed a significantly higher performance for histology compared to the examiners’ assessments (*p* < 0.001).

### 3.5. MCC by Lesion Class

**Figure 9:**
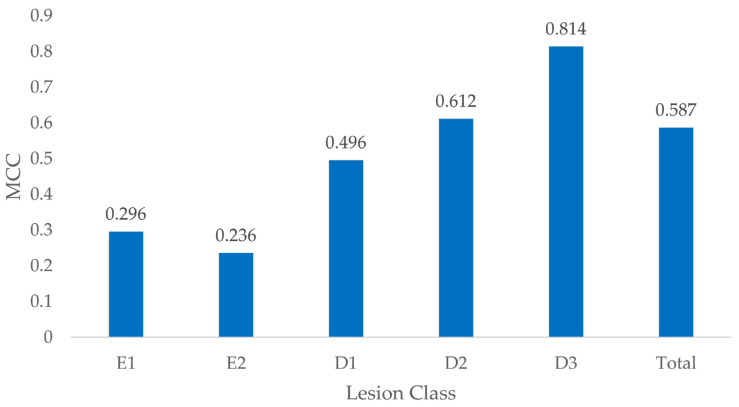
MCC by lesion class.

The MCC showed variation according to the penetration depth of the carious lesions, with the best performance observed for D3 lesions (0.814), whereas E2 lesions showed the least favorable result (0.236) (Figure 9). The aggregated MCC for all lesion categories was 0.587.

Testing for differences in MCC between different caries classifications revealed significant differences between all lesion classes (*p* < 0.008) except between E1 and E2 lesions (*p* = 1) (Table 6).

### 3.6. Gender Specific MCC

**Figure 10:**
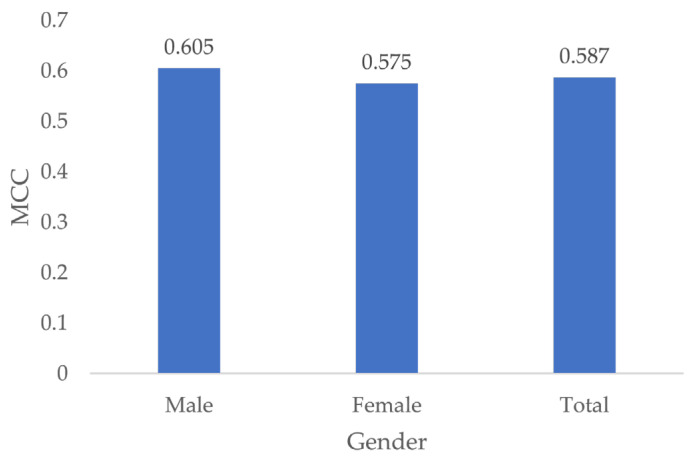
MCC by gender.

The MCC of male examiners was higher at 0.605 compared to the MCC of female examiners at 0.575 (Figure 10). However, testing for differences in MCC showed no significant effect of gender (*p* = 0.44).

### 3.7. MCC by Occupation

**Figure 11:**
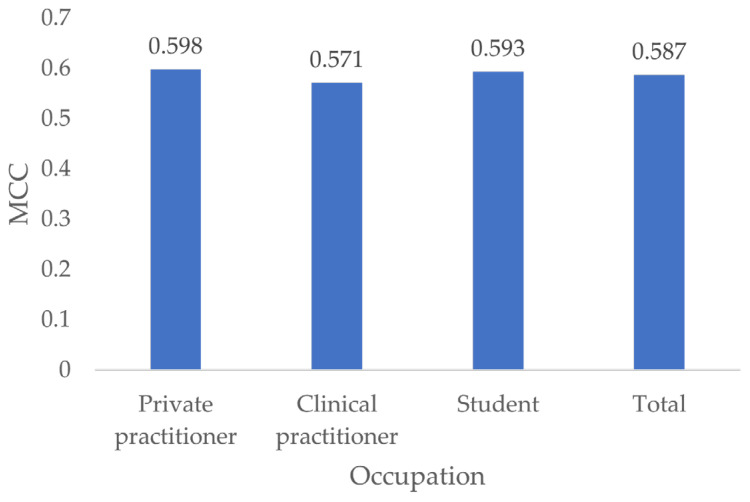
MCC by occupation.

Private practitioners had the highest MCC (0.595), followed by students (0.593) and clinical practitioners (0.571) (Figure 11).

Testing for differences in MCC showed no significant differences between all occupations (*p* ≥ 0.556).

### 3.8. MCC by Experience

**Figure 12:**
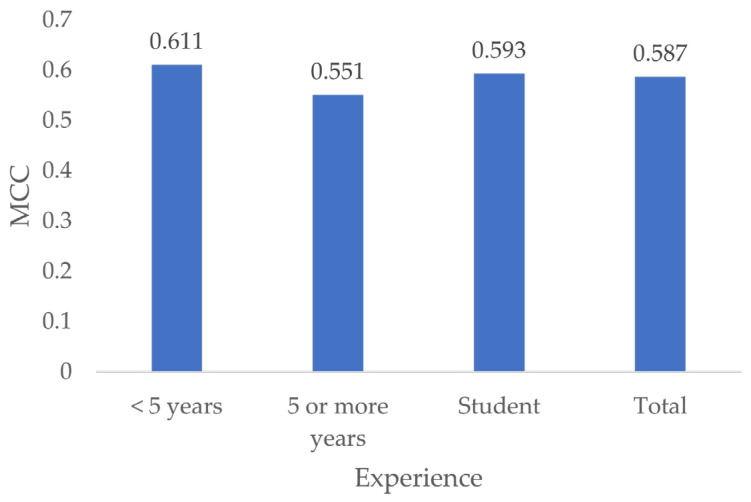
MCC by experience.

Dentists with less than 5 years of experience showed the best MCC (0.611), followed by students (0.593) and dentists with 5 or more years of experience (0.551) (Figure 12).

No significant differences were found by testing for differences in MCC according to experience (*p* = 1).

### 3.9. Influence of Eccentricity on MCC

**Figure 13:**
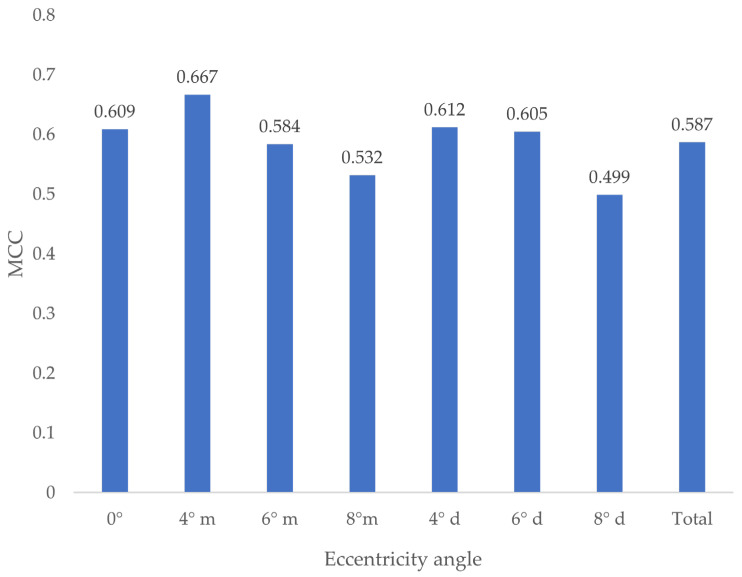
MCC by eccentricity angle. m = mesial-eccentric, d = distal-eccentric.

Different eccentricity angles resulted in different MCC values (Figure 13).

No statistically significant difference between the groups could be found (*p* ≥ 0.411).

### 3.10. Differentiation between Carious Lesions and Artifically Induced Lesions

**Figure 14:**
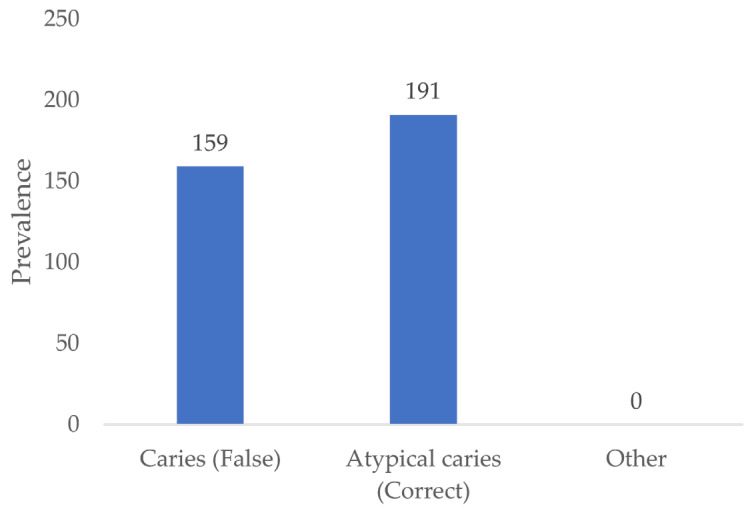
Assessment of artificially induced lesions.

Out of a total of 350 artificial defects presented, 159 defects (45.4%) were identified as carious lesions by all examiners and 191 defects (54.6%) were identified as atypical for caries (Figure 14).

### 3.11. Tooth Classification

**Figure 15:**
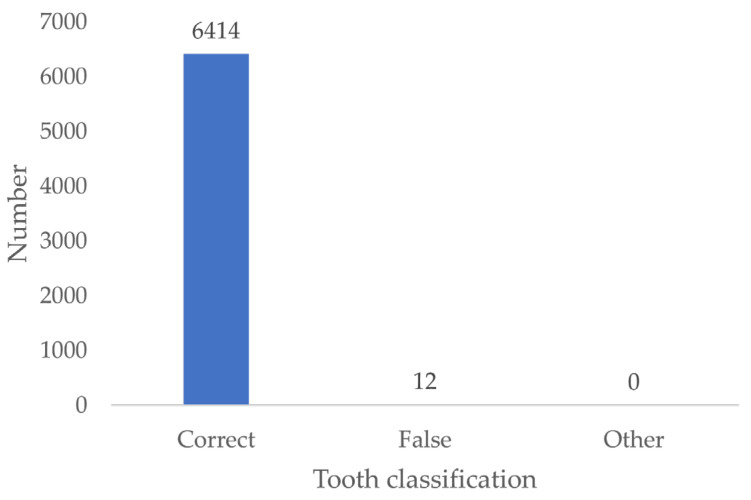
Tooth classification according to the FDI scheme.

The results indicate that 99.8% of the examiners correctly positioned the teeth depicted in the bitewing simulations according to the World Dental Federation (FDI) tooth numbering system (Figure 15).

## 4. Discussion

The European Medical Device Regulation (MDR) classifies medical imaging software as a medical device and, therefore, imposes several requirements on manufacturers to ensure safety and quality. Among other things, manufacturers are required to conduct a comprehensive clinical evaluation of their medical devices. As AI-based imaging software for caries diagnosis has been approved as a medical device, the underpinnings deserve scrutiny. The aim of this study was, therefore, to create a pool of histology-based radiographs to provide a scientifically sound testbed for such software. We are currently unaware of the existence of such a dataset.

In the context of fuzzy gold standards, several mitigation strategies have been proposed. One approach aims to supplement existing datasets with additional data from external sources [41]. By incorporating different perspectives, especially in cases where the gold standard may be imperfect, this strategy aims to improve the robustness of AI models and mitigate bias. The use of multiple diagnostic tests is also encouraged, as this can increase the transparency and reliability of diagnostic results [41]. Despite these efforts, the almost complete elimination of bias in AI-based dental caries diagnostics will, at least for an extended period, remain an elusive goal.

In general, in vitro studies provide a robust method for validating new caries diagnostic methods because they can refer to a reliable gold standard by means of histological analysis. Also, literature states that histological examination shall serve as the basis for a gold standard for the evaluation of new caries diagnostic methods [42]. Therefore, the ideal, albeit theoretical, method for evaluating diagnostic accuracy would be to first assess the diagnoses in vivo and then re-examine the same surfaces in vitro after tooth extraction using the histological gold standard [43]. However, logistical constraints and ethical considerations associated with invasive procedures, particularly the need for extraction, make this approach infeasible. Furthermore, it has been argued that differences between in vivo and in vitro results may cast doubt on the generalizability of in vitro data [43]. Nevertheless, previous studies have confirmed that no significant difference in the diagnostic accuracy of proximal carious lesions on digital radiographs can be demonstrated between in vivo and in vitro settings [44,45].

To further ensure the applicability of our results to the clinical situation, we attempted to create clinical simulations of the orofacial region on bitewing radiographs that are as realistic as possible. Nevertheless, given the complexity of the human body, accurate reproduction of anatomical structures remains difficult. To account for potential uncertainties, only findings within the coronal region were considered. This approach was intended to reduce possible distortions caused by the setup, particularly the fixation material. A limitation concerns the in vitro radiographs that did not consider external factors that could have influenced the accuracy of the radiographic diagnosis, such as the influence of metal artefacts, patient movement or incorrect positioning of the film holder on the analysis results. For reasons of standardization, all bitewing radiographs were taken on a single X-ray unit, to account for unintended variations.

For the purpose of disinfection and protection against dehydration, all extracted teeth were immersed in 1% tosylchloramide. Previous studies have shown that tosylchloramide has no discernible effect on tooth hard tissue [46,47,48,49]. A possible influence of tosylchloramide storage on the infiltration behavior of Technovit cannot be completely excluded, however it seems unlikely in view of the high success rate of histological preparations. All teeth were obtained from a variety of sources, including dental, oral and maxillofacial surgery practices and clinics. This diverse selection supports the assumption of a representative assortment of teeth across different population groups.

As already mentioned, histological examination serves as the most widely used gold standard for the validation of new caries diagnostic methods [42]. Its substantial diagnostic quality and value have been highlighted in many publications [50,51]. A major criticism of histological examinations is the frequent bisection of teeth through an arbitrary centerline [52]. This carries the risk of irreversibly destroying the presumed maximum extent of the carious lesion, thereby obscuring the true maximum depth. To overcome this, the incision was positioned anterior to the carious lesion, and the wet grinding technique was used to approach the maximum extent of the lesion. This approach ensured that the deepest carious extension was accurately identified with a high degree of confidence. The use of final multi-stage polishing ensured a consistent surface quality for subsequent expert analysis.

In our study, all 30 examiners showed a combined accuracy, sensitivity, specificity and AUC of 0.799, 0.565, 0.956 and 76.1, respectively, for the detection of carious lesions on bitewing radiographs. The literature shows a wide range of results. Kay and Knill-Jones observed a dentist sensitivity of 0.26 for the detection of dentin caries on in vitro bitewing radiographs [53]. Devlin et al. showed a sensitivity of 44% for enamel-limited lesions on bitewing radiographs among 23 examiners [54]. Mileman and van der Welle reported an AUC of 0.88 with a sensitivity of 0.54 and specificity of 0.97 for dentin caries on bitewing radiographs. Similarly, Peers et al. demonstrated a comparable sensitivity of 0.59 for the detection of dentin caries on bitewing radiographs [55]. It, therefore, can be assumed that the results of our study are consistent with the literature, as we also could demonstrate that carious lesion depth had a significant effect on the MCC of all examiners between all lesion classes, except between enamel-limited E1 and E2 lesions. We support the assumption that in vitro radiographs provide diagnostic quality parameters similar to studies using in vivo radiographs.

Our results also showed that, contrary to expectations, the eccentricity of the central X-ray beam up to 8°, whether mesial or distal, did not appear to have a significant effect on the examiner’s judgement of the presence or absence of caries. The lack of significant impact from minor eccentricities humbly suggests that clinicians may not need to be overly concerned about small variations in radiographic positioning when assessing for caries. Like our results, the study by Deprá et al. investigated the influence of the central opening angle on the diagnosis of secondary caries and also concluded that it had no influence [56]. On the other hand, Chadwick et al. investigated the influence of different central irradiation angles on visualization of proximal cavities in bitewing radiographs and found that lesions are typically diagnosed, often resulting in overtreatment [57]. However, as both comparative studies do not provide information on the size of the eccentricity examined, we are, to the best of our knowledge, the first study to provide results with tangible values.

In the present study, no significant effect of examiner experience could be demonstrated. The results, thus, contradict the findings of Geibel et al., which have shown that experienced examiners detect proximal lesions up to four times more frequently than less experienced examiners [58]. A plausible explanation for this difference could be that dental students and practicing dentists with less than five years of professional experience in our study took more time to analyze in vitro bitewing images than their colleagues with five or more years of clinical experience, as the time factor has been demonstrated to influence diagnostic accuracy significantly [31].

It was found that just over half (54.6%) of the artificial lesions were judged by the examiners to be atypical for caries, effectively distinguishing them from true carious lesions. This observation highlights the ability of human examiners to differentiate iatrogenic defects, such as those resulting from invasive treatments resulting from treatments of the adjacent teeth, from true caries cases, primarily through the assessment of lesion morphology. To the best of our knowledge, this study represents the first attempt to establish a framework for evaluating AI algorithms in this regard and to compare their performance with human judgement.

The empirical evaluation of binary classification tasks, such as the distinction between caries and healthy tooth structure, is subject of discussion. It must be noted that accuracy, as a metric, comes with the significant limitation of sensitivity to unbalanced datasets, potentially limiting the validity of the results. As the Fifth German Oral Health Study has already confirmed, caries prevalence is decreasing in all age groups, increasing the imbalance between carious and non-carious teeth on radiographs. Therefore, the suitability of accuracy to determine diagnostic quality must strongly be questioned [59]. Furthermore, Dinga et al. recommend completely omitting accuracy as sole criterion for evaluating clinical models, as it fails to take into account clinically relevant information [60]. Nevertheless, accuracy is still somewhat stubbornly used as the main parameter for performance evaluation in the literature. For the sake of comparability, we have included this metric, but explicitly point out its shortcomings. Positive predictive value (PPV), sensitivity, specificity and the F1 score, which is the harmonic mean of precision and recall, are commonly used parameters to evaluate binary classifiers [61]. However, these metrics assume that the “positive” class (in this case a detection of caries) is of primary interest, while true negatives are omitted in their calculation. Consequently, PPV, sensitivity and F1 scores are unaffected by variations in the number of true negatives, whether their value is extremely high or low. To overcome this limitation, we made use of Matthews correlation coefficient (MCC). MCC gives high values only when the predictions of all categories (true positives, true negatives, false positives and false negatives) show good performance, also considering the proportions of the positive and negative classes. As a result, the MCC is a statistically robust measure, even in the presence of unbalanced datasets.

## 5. Conclusions

The aim of this study was to establish a histology-based gold standard for the unbiased evaluation of AI-based caries detection systems on proximal surfaces in bitewing radiographs. Through meticulous in vitro simulations and histological analyses, we created a robust dataset to evaluate the performance of AI algorithms in caries detection and compare it to human judgement. Although AI promises to improve diagnostic accuracy and workflow efficiency, its effectiveness depends primarily on the quality of the training data and validation processes. Future research should be designed to accurately reflect the true performance of AI models using histological analysis as a benchmark. In doing so, we have laid the foundation for evaluating the real-world performance of AI systems, thereby advancing evidence-based dentistry. Ongoing advances in AI technology and regulatory frameworks require continuous refinement and validation of diagnostic tools to ensure patient safety and clinical effectiveness. The creation of a standardized database of reference histological specimens and associated radiographs could serve as a benchmark for the development and validation of new AI-based caries detection systems. This database would allow different AI systems to be compared and their performance tested against an established gold standard, helping to identify and develop the most accurate models. However, generating a histology-based dataset is time consuming and requires resources and equipment. Therefore, a simple histology-based implementation will not be readily available in the future. In addition, it remains to be seen whether newer intraoral caries detection techniques will provide higher sensitivity, which could serve as a solid basis for training dental AI systems. In conclusion, our study is an important step towards the creation of standardized evaluation protocols for AI-based caries detection, thereby promoting transparency, reliability and confidence in dental diagnostics.

## Figures and Tables

**Table 1:** Schematic overview of tooth dehydration and resin infiltration.

Time	Chemical Solution	Volume Ratio	Storage
Day 1	EtOH 70%/purif. H_2_O	100	50 mL plastic flacons
Day 2	EtOH 80%/purif. H_2_O	100	50 mL plastic flacons
Day 3	EtOH 90%/purif. H_2_O	100	50 mL plastic flacons
Day 4	EtOH 96%/purif. H_2_O	100	50 mL plastic flacons
Day 5 and 6	EtOH 99%	100	50 mL plastic flacons
Day 7	EtOH 99%/Technovit 7200 VLC	50/50	50 mL plastic flacons (in darkness)
Day 8	Technovit 7200 VLC	100	Snap-on lid tablet glass (in darkness)
Day 9	Technovit 7200 VLC	100	Snap-on lid tablet glass (in darkness)
Day 10	Technovit 7200 VLC	100	Snap-on lid tablet glass (in darkness)
Day 11	Technovit 7200 VLC	100	Snap-on lid tablet glass (in darkness)
Day 12	Technovit 7200 VLC	100	Snap-on lid tablet glass (in darkness)

purif. = purified, EtOH = ethanol.

**Table 2:** Caries classification scheme.

Classification of Caries	Carious Lesion Extension
E1	Caries limited to the outer half of the enamel
E2	Caries extending to the inner half of the enamel
D1	Caries in the outer third of dentin
D2	Caries in the middle third of dentin
D3	Caries in the dentinal third close to the pulp or up to the pulp

**Table 3:** Number of histologically confirmed carious lesions and their categorization according to the caries classification scheme.

Caries Classification	Proximal Surfaces	Percentage
E1	15	22.1%
E2	8	11.8%
D1	8	11.8%
D2	18	26.4%
D3	19	27.9%
	68	100%

**Table 4:** Examiner characteristics.

	Occupation			Experience		Gender	
	Private Practitioners	Clinicians	Students	<5 Years	≥5 Years	Male	Female
Occupation							
Private practitioners	10	-	-	6	4	6	4
Clinicians	-	10	-	5	5	5	5
Students	-	-	10	-	-	2	8

**Table 5:** Combined examiners’ performance metrics for caries detection.

Parameter	
Accuracy	0.799
Sensitivity	0.565
Specificity	0.956
PPV	0.896
NPV	0.765
F1 score	0.693
MCC	0.578
AUC	76.1

Note. AUC = area under the curve, MCC = Matthews correlation coefficient, NPV = negative predictive value, PPV = positive predictive value.

**Table 6:** Adjusted *p*-values for MCC comparison between lesion classes.

	Lesion Classification				
	E1	E2	D1	D2	D3
Lesion classification					
E1	-	1	<0.001 *	<0.001 *	<0.001 *
E2	1	-	<0.001 *	<0.001 *	<0.001 *
D1	<0.001 *	<0.001 *	-	0.008 *	<0.001 *
D2	<0.001 *	<0.001 *	0.008 *	-	<0.001 *
D3	<0.001 *	<0.001 *	<0.001 *	<0.001 *	-

Note. * Indicates significance (adjusted *p* < 0.05).

## Data Availability

The data presented in this study are available on request from the corresponding author due to ethical restrictions.

## Abbreviations

AI	Artificial intelligence
AUC	Area under the curve
EtOH	Ethanol
ICC	Intraclass correlation coefficient
NPV	Negative predictive value
MCC	Matthews correlation coefficient
PPV	Positive predictive value
ROC	Receiver operating characteristics
STARD	Standards for Reporting of Diagnostic Accuracy Studies
UV	Ultraviolet
